# High sPLA2-IIA level is associated with eicosanoid metabolism in patients with bacterial sepsis syndrome

**DOI:** 10.1371/journal.pone.0230285

**Published:** 2020-03-11

**Authors:** Nurul Saadah Ahmad, Toh Leong Tan, Khaizurin Tajul Arifin, Wan Zurinah Wan Ngah, Yasmin Anum Mohd Yusof

**Affiliations:** 1 Department of Biochemistry, Faculty of Medicine, Universiti Kebangsaan Malaysia, Jalan Ya’acob Latiff, Bandar Tun Razak, Cheras, Kuala Lumpur, Malaysia; 2 Department of Emergency Medicine, Hospital Canselor Tuanku Muhriz, Universiti Kebangsaan Malaysia Medical Centre, Jalan Ya’acob Latiff, Bandar Tun Razak, Cheras, Kuala Lumpur, Malaysia; 3 Biochemistry Unit, Faculty of Medicine and Defence Health, National Defence University of Malaysia, Sungai Besi Camp, Kuala Lumpur, Malaysia; Wayne State University, UNITED STATES

## Abstract

The aim of this study was to determine the association between secretory phospholipase A2 group IIA (sPLA2-IIA) and eicosanoid pathway metabolites in patients with bacterial sepsis syndrome (BSS). Levels of sPLA2-IIA, eicosanoids prostaglandin (PG)E2, PGD synthase were quantified in the sera from patients confirmed to have bacterial sepsis (BS; N = 45), bacterial severe sepsis/septic shock (BSS/SS; N = 35) and healthy subjects (N = 45). Cyclooxygenase (COX)-1 and COX-2 activities were analyzed from cell lysate. Serum levels of sPLA2-IIA, PGE2, and PGDS increased significantly in patients with BS and BSS/SS compared to healthy subjects (p<0.05). COX-2 activity was significantly increased in patients with BS compared to healthy subjects (p<0.05), but not COX-1 activity. Binary logistic regression analysis showed that sPLA2-IIA and PGE2 were independent factors predicting BSS severity. In conclusion, high level of sPLA2-IIA is associated with eicosanoid metabolism in patients with BSS.

## 1. Introduction

Bacterial sepsis syndrome (BSS) may cause organ failure and eventual death due to dysregulation of the systemic host response following a bacterial infection [1]. This syndrome involves physiological, pathological and biochemical disturbances in the host body system. Sepsis accounts for more than 1.5 million cases presented to emergency departments (ED) in the United States annually and is expected to increase in the coming years [2–4]. Sepsis cases on mainland China account for approximately one-fifth of those occurring in the world’s population [5]. In Malaysia, sepsis was documented as the fourth leading cause of death and accounts for 13% of other causes of death [6]. A rapid diagnosis of sepsis ensures a timely and a more effective treatment to avoid deterioration in organ function [7–10]. The classical diagnostic method is based on signs of an inflammatory response and microbial testing. Despite that, a culture-dependent diagnosis of infection is time-consuming, it is still considered the gold standard assessment. Hence, a panel of inflammatory markers rather than a single biomarker has been suggested to provide a more rapid way to rule BSS in or out [11–13]. Early recognition and intervention of sepsis through a rapid diagnosis are believed to be the key to improve survival rates [12].

A systematic review conducted by Pierrakos & Vincent (2010) [14] described five sepsis biomarkers with >90% sensitivity and specificity values. The biomarkers include interferon-induced protein 10 [15], neutrophil CD11b [16,17], interleukin-12 (IL-12) [18], CD64 [19,20] and secretory phospholipase A2 group IIA (sPLA2-IIA) [19,21,22]. All of these five sepsis biomarkers play important roles in triggering the sepsis inflammatory pathway, but only sPLA2-IIa can be used to differentiate bacterial infection in adults [19–25]. Other biomarkers, such as serum procalcitonin and C-reactive protein, also have a similar property, but sPLA2-IIA is more specific and sensitive for diagnosing BSS [19,26–28].

One of the most common inflammation pathways related to sepsis is the eicosanoid pathway. Eicosanoids are not only involved in the inflammation process but also enhanced bactericidal activity [29–33]. Based on their central pathophysiological effects and modulatory function on inflammation, eicosanoids serve an important role as inflammatory markers [34,35]. During infection, activated sPLA2-IIA will hydrolyze fatty acids from the cell’s membrane phospholipid to liberate arachidonic acids and lysophospholipids. The cyclooxygenase (COX) converts arachidonic acids to various types of prostaglandins (PGs), prostacyclins and thromboxanes [35].

Until now, no study has combined sPLA2-IIA and eicosanoids in relation to the development of BSS. Even though the role of sPLA2-IIA has been described extensively in bacterial infection and other inflammatory diseases, the pathophysiological mechanism of sepsis with regards to sPLA2-IIA and eicosanoid pathway is still not fully understood. Therefore, this study determined the association between sPLA2-IIA and eicosanoids in patients with BSS. We hypothesised that there is association between sPLA2-IIA and eicosanoids in patients with BSS. The contribution of this study is important as a basis for the progression of BSS as well as understanding how sPLA2-IIA relates to the sepsis inflammatory mechanism in patients with a bacterial infection, and eventually assists medical practitioners in providing better sepsis management and early antibiotic treatment through the discovery of bacterial sepsis biomarkers.

## 2. Materials and methods

This study was conducted from March 2014 to July 2016 after obtaining approval from Universiti Kebangsaan Malaysia (UKM) Research Ethics Committee (Ethics code: FRGS/1/2014/SKK01/UKM/03/3) (S1 Fig).

### 2.1. Recruitment of research subjects

Written consent was obtained from all subjects or their legal substitute prior to enrolment. This single-centred prospective observational study consisted of consenting patients who presented to the ED of UKM Medical Centre, which is a tertiary teaching hospital with approximately 72,000 visits annually. All patients with a suspected infection [36] and positive systemic inflammatory response syndrome (SIRS) criteria aged ≥18 years old were recruited. Eligible subjects were classified into three different outcomes of Bacterial Sepsis (BS), Bacterial Severe Sepsis/Septic Shock (BSS/SS) and healthy subjects. BS was defined as a patient with a minimum of two positive SIRS criteria and a bacteria-positive culture. BSS/SS was defined as BS with organ failure [37] or bacterial septic shock, which was defined by the presence of hypotension (systolic blood pressure <90 mmHg) despite adequate fluid resuscitation [37,38]. Exclusion criteria were patients that were partially treated with antibiotics for >3 days, patients with cancer, immunodeficiency, auto-immune diseases or those taking non-steroidal anti-inflammatory drugs or steroids. Healthy subjects negative for SIRS and no evidence of infection were recruited as controls.

### 2.2. Sample collection & storage

The initial 3 ml of blood was taken from each eligible subject within 1 to 6 hours of arrival to ED, before any treatment given. The blood was transferred to vacutainer tubes containing clotting agents and a serum separator gel. The tube was shaken gently several times and left in an upright position at room temperature for 15–30 min to allow complete blood clotting. The samples were centrifuged at 1,300× g for 10 min. The supernatant containing serum was transferred to secondary tubes. Serum samples and residual blood cells were stored frozen at −20°C until further analysis. Relevant cultures and serology tests were carried out for all patients by our in-house Laboratory Diagnostic & Pathology Department before the antibiotic treatment. Positive culture results were recorded. Blood samples from positive-result bottles were stained with Gram stain. Appropriate antibiotics were administered to the patients within 1 hour of the sepsis diagnosis.

### 2.3. Determination of sPLA2-IIA and eicosanoids in serum and residual blood cells

To determine the association between sPLA2-IIA and eicosanoids in patients with BSS, we first measured the levels of sPLA2-IIA and eicosanoids.

Serum levels of sPLA2-IIA, PGE2 and PGDS were detected using commercially available immunoassay kits, according to the manufacturer’s instructions: the sPLA2-IIA (human type IIA) Enzyme Immunometric Assay Kit (Cayman Chemical, Ann Arbor, MI, USA), PGE2 EIA Kit-Monoclonal (Cayman Chemical) and Human PGDS ELISA Kit (Elabscience, Beijing, China). All assays were performed in duplicates, and the unknown levels were determined against a standard curve of each assay. Absorbance was measured between 405 and 450 nm (sPLA2-IIA and PGE2 level) and 450 nm (PGDS level).

For COX activities, residual blood cells comprising of leukocytes, platelets and erythrocytes were thawed and sonicated in 500 μl of cold buffer (0.1 M Tris-HCl, 1 mM EDTA and 0.05% Triton X-100) for 30 min. The mix was then centrifuged at 4°C and 10,000 ×g for 15 min, and the cell lysate (supernatant part) was transferred to a secondary tube. The cell lysate was assayed directly or stored frozen at −20°C for up to 1 month.

COX activities were assayed in duplicates according to the manufacturer’s protocol (Cayman Chemical). Specific inhibitors were used to distinguish between COX-1 and COX-2 activities. DuP-697 is a potent and time-dependent inhibitor of COX-2, whereas SC-560 is a potent and selective COX-1 inhibitor. Both inhibitors are provided by the manufacturer (Cayman Chemical), and samples incubated with DuP-697 or SC-560 eliminates all COX-2 or COX-1 activity, respectively. Thus, specific COX-1 and COX-2 activity can be calculated by using the specific inhibition. Absorbance was measured at a wavelength of 590 nm.

### 2.4. Statistical analysis

All statistical analyses were performed using SPSS version 22.0 for Windows software (SPSS Inc., Chicago, IL, USA. Continuous data are presented as mean ± standard error of the mean, and categorical data are expressed as a percentage. Analysis of normality was performed using the Kolmogorov–Smirnov test. Differences between the groups in the *in vivo* and *in vitro* studies were tested using analysis of variance for normally distributed continuous variables. A p-value < 0.05 was considered significant. Levels of sPLA2-IIA and eicosanoid metabolites (dependent variable) were tested against healthy, BS and BSS as outcomes (independent variable) using binary logistic regression (BLR). A multiple linear regression (MLR) analysis was used to identify the factors related to sPLA2-IIA level (dependent variable).

## 3. Results

### 3.1 Characteristics of study population

**Fig 1:**
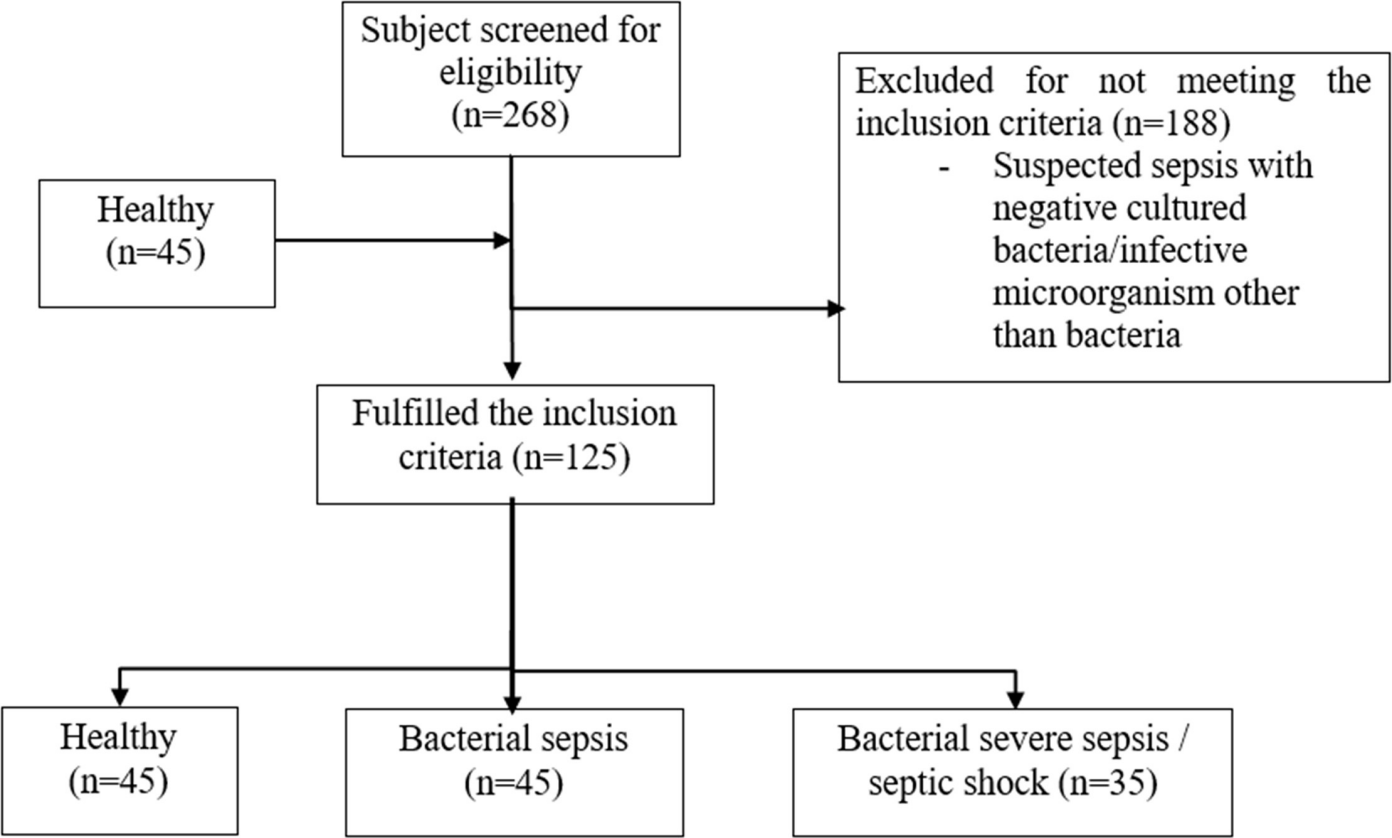
Recruitment of research subjects.

We screened 268 patients who presented to the ED with suspected sepsis from March 2014 to July 2016. With the study’s strict recruitment criteria, only 80 patients were eligible for the study (Fig 1) following culture-confirmed bacterial infections. Forty-five patients had BS and 35 had BSS/SS. A total of 188 patients were excluded due to negative cultures. Forty-five healthy subjects were recruited for this study. The demographic data of the recruited subjects are shown in Table 1. Age, gender, race and source of infection were not related to BS or BSS/SS. The Systemic Inflammatory Response Syndrome (SIRS) criteria (heart rate, respiratory rate, temperature and total white blood count) increased significantly in the BS and BSS/SS groups compared to the healthy group. There were more Gram-negative bacteria (N = 50) as compared to Gram-positive (N = 30) bacteria detected in blood culture isolation. The mean of sPLA2-IIA level in Gram-positive bacteria and Gram-negative bacteria were 18.18 ± 12.67 and 21.37 ± 13.58 ng/ml; respectively. The bacterial aetiology as detected by cultures is shown in S1 Table. Of the 33 bacteria species, *Escherichia coli* was the most frequently isolated bacteria from the cultures.

**Table 1:** Demographic and clinical characteristics of healthy subjects, bacterial sepsis (BS) and bacterial severe sepsis/ septic shock (BSS/SS).

	Healthy	^†^BS	^††^BSS/SS	*p* value
	(n = 45)	(n = 45)	(n = 35)	
**Age (mean ± SEM)**	49.1 ± 16.5	56.6 ± 18.3	62.8 ± 15.2	0.050 ^a^
**Gender, n(%)**				
Male	26 (57.8)	28 (62.2)	22 (62.9)	0.872 ^d^
Female	19 (42.2)	17 (37.8)	13 (37.1)	
**Race, n(%)**				
Malay	44 (97.8)	26 (57.8)	13 (37.9)	0.511 ^d^
Chinese	1 (2.2)	13 (28.9)	15 (42.9)	
Indian	0 (0.0)	4 (8.9)	3 (8.6)	
Others	0 (0.0)	2 (4.4)	2 (11.4)	
**SIRS Criteria (mean ± SEM)**				
Heart rate (per minute)	74 ± 15	109 ± 16 ^b^	113 ± 21 ^b^	<0.001 ^a^*
Respiratory rate (per minute)	18 ± 3	21 ± 5 ^b^	25 ± 8 ^b^^,^^c^	<0.001 ^a^*
Temperature (°Celcius)	36.5 ± 0.6	38.1 ± 1.1 ^b^	38.2 ± 1.1 ^b^	<0.001 ^a^*
Total White Blood Count(per microliter)	8.1 ± 1.6	15.1 ± 7.5 ^b^	15.0 ± 11.2 ^b^	<0.001 ^a^*
**Source of infection, n(%)**				
Respiratory	N/A	9 (20.0)	4 (20.0)	0.605 ^d^
Skin		5 (11.1)	4 (11.4)	
Gastrointestinal		5 (11.1)	8 (22.9)	
Urinary tract		10 (22.2)	5 (14.3)	
Blood/Catheter		7 (15.6)	6 (17.1)	
Central nervous system		2 (4.4)	0 (0.0)	
Musculoskeletal		7 (15.6)	5 (14.3)	
**Level of inflammatory markers (mean ± SEM)**				
sPLA2-IIA (ng/ml)	1.0 ± 0.1	18.3 ± 1.7 ^b^	22.6 ± 2.5 ^b^	<0.001 ^a^*
PGE2 (ng/ml)	591.1 ± 107.7	2121.0 ± 245.8 ^b^	2127.5 ± 283.7 ^b^	<0.001 ^a^*
PGDS (ng/ml)	4.1 ± 0.3	8.7 ± 0.7 ^b^	6.8 ± 0.7 ^b^	<0.001 ^a^*
**COX activities**				
COX-1 (nmol/unit/ml)	64.1 ± 4.9	33.3 ± 4.9 ^b^	46.3 ± 5.9 ^b^	<0.001 ^a^*
COX-2 (nmol/unit/ml)	42.5 ± 4.7	61.7 ± 4.6 ^b^	55.6 ± 6.4 ^b^	0.021

Abbreviations: SEM standard error of mean, sPLA2-IIA secretory phospholipase A2 group IIA, PGE2 prostaglandin E2, PGDS prostaglandin D synthase, COX cyclooxygenase enzyme.

^a^ ANOVA test (for continuous data)

^b^ post-hoc Bonferroni; p<0.05 as compared to healthy

^c^ post-hoc Bonferroni; p<0.05 as compared to BS

^d^ Chi-Square test (for categorical data)

† Bacterial sepsis

†† Bacterial severe sepsis/septic shock

* Significant difference at p<0.05

#### 3.1.1. Level of inflammatory markers in patients with bacterial sepsis syndrome

The levels of sPLA2-IIA, PGE2 and PGDS increased significantly in the BS and BSS/SS groups compared to the healthy subjects (p < 0.05). COX-1 activity decreased, while COX-2 activity increased significantly in patients with BS compared to the healthy subjects (p < 0.05). No significant differences in any other parameters were observed among the patients (Table 1).

#### 3.1.2. Prediction of sepsis syndrome severity using sPLA2-IIA and the eicosanoid metabolites

For Binary Logistic Regression (BLR), univariate analysis was performed. sPLA2-IIA and eicosanoids were found to contribute significantly to BSS (Table 2). The overall multivariate BLR model predictive ability for BS was 98%; Nagelkerke R Square (NRs) = 0.90. The model had an excellent goodness-of-fit with a Hosmer-Lemershow Test (HLT) value of 0.48, where z = -5.271 + 1.426 (sPLA2-IIA) + 0.001 (PGE2). The probability of BS = e^-5.271 + 1.426(sPLA2-IIA) + 0.001(PGE2).^

**Table 2:** Factors associated with sepsis syndrome patients (using Binary Logistic Regression).

	BS		95% CI for OR		BSS/SS		95% CI for OR	
Dependent variables	*p* value	OR	Lower	Upper	*p* value	OR	Lower	Upper
**Univariate**								
sPLA2-IIA (ng/ml)	0.001*	3.513	1.850	6.670	0.001	3.600	1.895	6.838
PGE2 (ng/ml)	0.001*	1.137	1.001	1.932	0.001	1.139	1.001	1.942
PGDS (ng/ml)	0.001*	1.941	1.467	2.569	0.001	1.727	1.310	2.277
COX-1 (nmol/unit/ml)	0.001*	0.973	0.960	0.987	0.031	0.986	0.973	0.999
COX-2 (nmol/unit/ml)	0.008*	1.018	1.005	1.032	0.054	1.014	1.000	1.029
**Multivariate**								
sPLA2-IIA (ng/ml)	0.002*	1.426	1.696	10.212	0.001*	2.314	1.875	10.769
PGE2 (ng/ml)	0.043*	0.001	1.000	1.002	0.035*	0.002	1.000	1.004

*Significant difference at *p*<0.05

For BSS/SS, the overall multivariate BLR model predictive ability was 98%; Nagelkerke R Square (NRs) = 0.98. The model had an excellent goodness-of-fit with a Hosmer-Lemershow Test (HLT) value of 0.96, where z = -10.511 + 2.314 (sPLA2-IIA) + 0.002 (PGE2). The probability of BSS/SS = e^-10.511 + 2.314(sPLA2-IIA) + 0.002(PGE2).^ sPLA2-IIA and PGE2 were independent predictors of BSS severity.

Multiple Linear Regression was then performed to evaluate the association of sPLA2-IIA and eicosanoids and SIRS among BSS patients. Higher sPLA2-IIA was independently associated with PGE2 (*b* = 0.002, P = 0.001) and PGDS (*b* = 1.3, p = 0.001) in the Multiple Linear Regression (MLR) analysis that included the significant univariate variables (Table 3). This model suggests that sPLA2-IIA contributed about 22% to activation of the eicosanoid pathway (R^2^ = 0.22), and was also independently associated with the SIRS criteria, such as heartbeat (*b* = 0.19, p = 0.001), temperature (*b* = 3.37, p = 0.001) and white blood cell count (*b* = 0.35, p = 0.008) and contributed 37% to the SIRS criteria in our study sample (R^2^ = 0.37).

**Table 3:** Correlation, univariate and multivariate linear regression of sPLA2-IIA and eicosanoid metabolites and SIRS.

Dependent variables	R^2^	*^b^* (CI)	*p* value ^a^
**Univariate**			
Eicosanoid metabolites			
- PGE2 (ng/ml)	0.088	9.12 (5.74, 12.50)	0.001*
- PGDS (ng/ml)	0.153	4.49 (0.15, 8.83)	0.043*
- COX-1 (nmol/unit/ml)	0.028	16.46 (12.33, 20.59)	0.001*
- COX-2 (nmol/unit/ml)	0.019	10.27 (5.65, 14.90)	0.001*
SIRS Criteria			
- Heart rate (beats/min)	0.269	-16.2 (-25.45, -6.90)	0.001*
- Respiratory rate (breaths/min)	0.115	-2.51 (-11.13, -6.10)	0.565
- Temperature (˚C)	0.233	-196.86 (-266.58, -127.14)	0.001*
- Total white blood count (μl)	0.122	6.09 (1.69, 10.50)	0.007*
			
**Multivariate**			
Model 1 (backward) sPLA2-IIA & eicosanoid metabolites	0.22	6.43 (1.32, 11.54)	0.014*
- PGE2		0.002 (0.001, 0.004)	0.001*
- PGDS		1.272 (0.726, 1.818)	0.001*
Model 2 (backward) sPLA2-IIA & SIRS criteria	0.37	-135.44 (-204.40; -66.65)	0.001*
- Heart rate		0.19 (0.08, 0.29)	0.001*
- Temperature		3.37 (1.42, 5.32)	0.001*
- Total white blood count		0.35 (0.09, 0.61)	0.008*

^a^ dependent variable sPLA2-IIA

*Significant difference at *p*<0.05

*b* crude regression coefficient

The model reasonably fits well. Model assumptions are met. There is no multicollinearity problem.

## 4. Discussion

The pathophysiological mechanism of sepsis is complex and is still not fully understood [1,39]. The role of sPLA2-IIA has been described extensively in other inflammatory diseases, such as atherosclerosis [40], lung cancer [41], ocular surface drought [42], rheumatoid arthritis [43] and neurodegenerative diseases [44]. In addition, sPLA2-IIA has bactericidal activity [45,46]. However, understanding of the role of sPLA2-IIA and its association with arachidonic acid metabolism is limited in BS cases.

In the present study, we observed that sPLA2-IIA level, along with the levels of PGE2, PGDS and COX-1 and COX-2 activities, differentiated patients with BS and BSS/SS from the healthy controls. This confirms sPLA2-IIA was significantly related to the eicosanoid metabolism during bacterial infection. Our findings are in line with previous studies suggesting that sPLA2-IIA [14,19,23], PGE2 [47], PGDS [48,49] and COX-2 [50] are biological markers, not only in inflammation response-related diseases but also in patients with BS. According to Tan et al. [19], sPLA2-IIA potentially provides high sensitivity and specificity for diagnosing BS infections. The bacterial destruction mechanism of sPLA2-IIA is explained by degradation of the bacterial cell membrane which has a phospholipid-rich zone. Positively charged (cationic) sPLA2-IIA enhances the effectiveness of the enzyme to approach and attach to the surface of the negatively charged (anionic) bacterial cells [51]. This special characteristic suggests that sPLA2-IIA is a potential sepsis biomarker, particularly for differentiating a bacterial infection from a non-bacterial infection [19,22,24]. Multiple studies have explored the antimicrobial activities of sPLA2-IIA towards Gram-stained bacterial species [52,53]. The sPLA2-IIA level is found to be lower in Gram-positive bacterial infection, suggesting that this group of bacteria are more sensitive to the bactericidal effect of sPLA2-IIA. Towards the Gram-positive bacteria tested thus far, the global cationic properties of sPLA_2_-IIA are necessary for optimal binding to intact bacteria and penetration of the multi-layered thick cell wall. Hence, as our findings suggest a combination of a higher sPLA2-IIA level together with the host’s defense systems are required to kill Gram-negative bacteria [52,54].

Due to their natural role in the pathophysiology of inflammation and overall contribution to modulating the inflammatory response, eicosanoids are also considered potential inflammatory markers of BSS [34,35,47,55]. Previous studies have shown that cyclooxygenase-generated PGE2 is a metabolite that can be used to differentiate BS from healthy subjects [47]. PGDS level has been reported in diseases related to general inflammation, such as bronchial asthma, pre-eclampsia and ulcerative colitis [48]. However, the knowledge of its role in inflammation due to infection is very limited. PGDS is involved in enhancing the pro-inflammatory response of macrophages and subsequent activation of neutrophils [49]. Previous studies have also shown a contrary action between COX-1 and COX-2 activities during the inflammatory response, especially during organ failure. Bruegel et al. [47] reported a significant decrease in thromboxane levels (another metabolite other than PGs) in a sepsis group compared to a healthy control group. Their findings were also in line with another study by Höcherl et al. [56], who showed that COX-2 expression increases while COX-1 expression decreases when rats were treated with LPS. They argued that the dysregulation of COX-1 and COX-2 activities occur due to organ damage resulting from endotoxemia. This is possibly due to the diminished production of COX-1-derived prostanoids during the inflammatory response [56].

In the present study, there were no difference in the level of sPLA2-IIA or eicosanoids between the patients with BS and BSS/SS, suggesting that the markers are not useful to distinguish sepsis severity. Surprisingly, our findings were contrary to other studies that correlated increased PLA2 catalytic activity in circulation with tissue injury severity [57,58]. The concentration of eicosanoid metabolites increases significantly with sepsis severity [47]. However, this phenomenon can be explained by compensatory anti-inflammatory response syndrome (CARS). CARS causes a regulatory imbalance in pro- and anti-inflammatory mediators during severe infections [59,60], which eventually leads to immunosuppression and organ failure as described for severe sepsis and septic shock [61]. Increased levels of anti-inflammatory mediators, such as interleukin (IL)-4, IL-10 and IL13 are also associated with decreased PG levels during prolonged inflammation processes [62]. However, this finding may need further evaluation with a larger sample.

A study by Ostrowski et al. [63] showed that the association between biological markers of endothelial injury and hypocoagulability among patients with severe sepsis was significant but accounted for only 10% of the overall hypocoagulability process. Their results were supported by other studies showing a poor association between endothelial cell activation markers and sepsis severity, organ failure and death [64]. The similarities in all of these studies were that sepsis biomarkers from human blood samples were analysed, and the results showed significant but rather poor relationships between the biological markers. These associations may be partly explained by the septic pathophysiology itself which involves various inflammatory mechanisms and mediators [64,65].

Although the present study obtained valuable results regarding sPLA2-IIA in bacterial sepsis, there were several limitations. First, this study only reflects infection caused by bacteria. It does not cover sources of infection from other microorganisms such as viruses, parasites and fungus. Second, this database was relatively old (patients between 2014 and 2016) and some clinical management strategies have been improved for sepsis recently. Third, larger sample size is needed to assess performance of sPLA2-IIA and eicosanoid as panel of diagnostic biomarkers.

Overall, the level of sPLA2-IIA and eicosanoid pathway metabolites could be used to differentiate between groups of patients with sepsis and healthy subjects. The association between sPLA2-IIA and eicosanoids indicate the complexity of sepsis pathophysiology and explained the interactions among various pro-inflammatory mediators, such as other cytokines and chemokines, during sepsis. These findings are expected to assist medical practitioners in providing better sepsis management and early antibiotic treatment.

## 5. Conclusions

In summary, sPLA2-IIA is associated with eicosanoids in patients with BS and BSS/SS, reflected clinically as SIRS or CARS, which are the sequelae of BSS. Combined measurement of sPLA2-IIA and eicosanoid metabolite levels may potentially be used as a panel of biomarkers for early diagnosis and prognosis for BSS. Hence, it is important for further studies to explore the potential of sPLA2-IIA as a biomarker for BSS diagnosis and prognosis.

## Supporting information

S1 Fig Study flow chart. (PDF)

S1 Table Bacterial etiology as confirmed via blood culture and sensitivity test. (PDF)
