# Supplementary material for: High sPLA2-IIA level is associated with eicosanoid metabolism in patients with bacterial sepsis syndrome
Source: PLoS One. 2020 Mar 11;15(3):e0230285. doi: 10.1371/journal.pone.0230285 (PMC7065791; doi:10.1371/journal.pone.0230285)
Supplement: S1 Fig — (PDF) [file pone.0230285.s001.pdf]

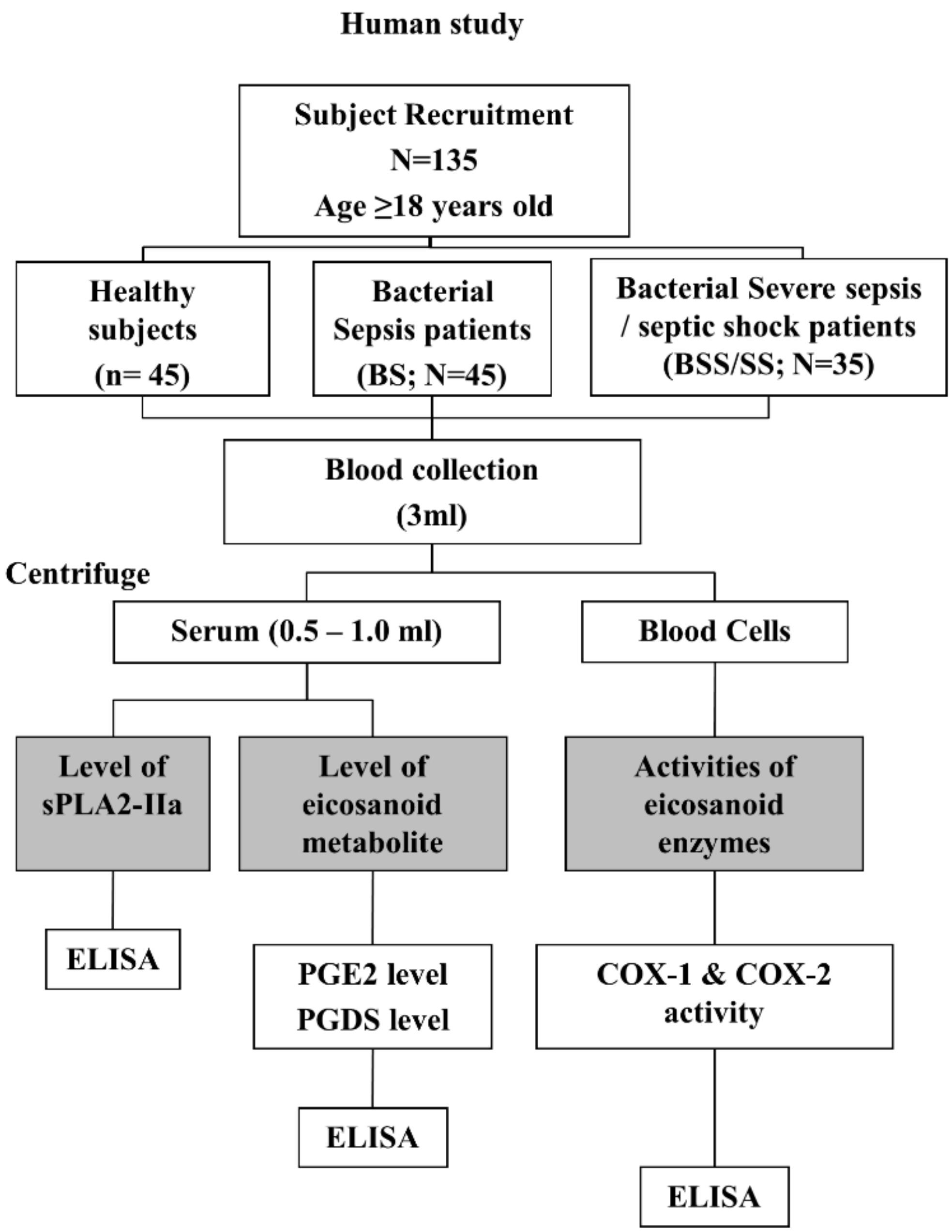

S1 Fig. Study flow chart. This study consists of three groups of research subjects. The aim of this study was to determine the association between secretory phospholipase A2 group IIA (sPLA2-IIA) and eicosanoid pathway metabolites in patients with bacterial sepsis syndrome (BSS).
