# Supplementary material for: High sPLA2-IIA level is associated with eicosanoid metabolism in patients with bacterial sepsis syndrome
Source: PLoS One. 2020 Mar 11;15(3):e0230285. doi: 10.1371/journal.pone.0230285 (PMC7065791; doi:10.1371/journal.pone.0230285)
Supplement: S1 Table — (PDF) [file pone.0230285.s002.pdf]

S1 Table. Bacterial etiology as confirmed via blood culture and sensitivity test.

Bacterial species	Frequency	Gram-stained Bacteria
Gram-negative bacterial species (N=50)		
Escherichia coli	23	Gram-negative
Pseudomonas aeruginosa	6	Gram-negative
Enterobacter sp.	6	Gram-negative
Proteus sp.	7	Gram-negative
Klebsiella sp.	5	Gram-negative
Gram negative rods	3	Gram-negative
Bacteroides sp.	2	Gram-negative
Burkholderia cepacia	2	Gram-negative
Acinetobacter sp.	2	Gram-negative
Morganella_morganii	3	Gram-negative
Enterococcus sp.	2	Gram-negative
Coynebacterium sp.	1	Gram-negative
Aeromonas hydrophila	1	Gram-negative
Rhodococcus sp.	1	Gram-negative
Serratia sp.	1	Gram-negative
Bacteroides fragilis	1	Gram-negative
Enterobacter cloacae	1	Gram-negative
Chlamydia pneumoniae	1	Gram-negative
Moraxella sp.	1	Gram-negative
Pseudomonas sp.	1	Gram-negative
Gram-positive bacterial species (N=30)		
Staphylococcus coagulase negative	8	Gram-positive
Streptococcus B, Haemolytic group G	7	Gram-positive
Staphylococcus aureus	5	Gram-positive
Methicillin Resistant Staphyloccus aureus	5	Gram-positive
Leptospira sp.	3	Gram-positive
Gram positive cocci	2	Gram-positive
Mycobacterium tuberculosis	1	*N/A
Staphylococcus pyrogen	1	Gram-positive
Streptococcus viridans	1	Gram-positive

*N/A: non-applicable.
